# Prognostic role of quantitative [18F]FDG PET/CT parameters in adrenocortical carcinoma

**DOI:** 10.1007/s12020-024-03695-6

**Published:** 2024-02-21

**Authors:** Wiebke Schlötelburg, Philipp E. Hartrampf, Aleksander Kosmala, Carmina T. Fuss, Sebastian E. Serfling, Andreas K. Buck, Andreas Schirbel, Stefan Kircher, Stefanie Hahner, Rudolf A. Werner, Martin Fassnacht

**Affiliations:** 1 Department of Nuclear Medicine, University Hospital Würzburg, Oberdürrbacher Straße 6, 97080 Würzburg, Germany; 2 Division of Endocrinology and Diabetes, Department of Medicine I, University Hospital Würzburg, Würzburg, Germany; 3 Institute for Pathology, University of Würzburg, Würzburg, Germany; 4 Division of Nuclear Medicine and Molecular Imaging, The Russell H. Morgan Department of Radiology and Radiological Science, The Johns Hopkins University School of Medicine, Baltimore, MD USA; 5 Goethe University Frankfurt, University Hospital, Department of Nuclear Medicine, Clinic for Radiology and Nuclear Medicine, Frankfurt, Germany

**Keywords:** Endocrine, ACC, Survival, SUV_max_, Treatment-naïve

## Abstract

**Purpose:**

We aimed to evaluate the prognostic potential of baseline [^18^F]FDG PET/CT for overall survival (OS) in patients with adrenocortical carcinoma (ACC).

**Methods:**

We performed a retrospective analysis of 67 treatment-naïve ACC patients with available [^18^F]FDG PET/CT at time of initial diagnosis. Pretherapeutic PETs of primary tumors were manually segmented and quantitative parameters (maximum/mean/peak standardized uptake value (SUV_max/mean/peak_), metabolic tumor volume (MTV) and tumor lesion glycolysis (TLG, defined as TV*SUV_mean_) were derived. Based on a visual read, absence (M0) or presence of metastatic disease (M1) were evaluated. Kaplan–Meier and Cox regression analyses were used to determine the prognostic value of the above mentioned markers on overall survival adjusted for established prognostic markers.

**Results:**

24/67 patients (36%) presented with M0 based on PET/CT, while the remaining 43/67 (64%) had M1-status. 32/67 patients died during follow-up and median OS was 48 months. In 12% of patients FDG-PET detected additional metastatic lesion not clearly visible by CT only. In univariable analysis, all quantitatively derived PET parameters failed to reach significance (*P* *≥* 0.1), and only PET/CT-based M1-status and Ki-67 were associated with increased mortality (M1: *HR* 13.89, *95% CI* 4.15–86.32, *P* < 0.001; Ki-67 HR 1.29, *95% CI 1.16*–*1.42; P* < *0.0001*). Using multivariable Cox regression analyses, M1-status (*HR* 9.69, *95% CI* 2.82–60.99) and Ki-67 index (*HR* 1.29, *95% CI* 1.13–1.04*; P* < 0.05) remained significant associated with OS.

**Conclusion:**

In treatment-naïve ACC patients, the quantitative PET parameter failed to predict OS, but presence of metastases detected by [^18^F]FDG PET/CT and Ki-67 index were independently associated with shorter OS. Therefore, a simple visual PET-based read-out is of prognostic value at initial diagnosis, while time-consuming PET-based quantification can be omitted.

## Introduction

Adrenocortical carcinoma (ACC) is a rare endocrine malignancy (incidence of 0.7–2 cases per million population/year) [1, 2]. Five-year survival rates are heterogeneous, ranging from 0% to 80%, and depend mainly on tumor stage, resection status, hormone excess and proliferative activity/tumor grade [3–5]. Reliable non-invasive prognosticators for patients, such as imaging parameters, would be favorable, as they would allow treatment to be modified or intensified early in the course of the disease.

To date, the European Network for the Study of Adrenal Tumors (ENSAT) tumor staging system is commonly used to stage patients with ACC [3, 5, 6]. Recent modifications have made this standardized framework also applicable to widespread metastatic disease [7, 8]. Not surprisingly, recent years have seen an increased use of the glucose consumption-reflecting positron emission tomography/computed tomography (PET/CT) biomarker 2-[^18^F]fluoro-2-deoxy-D-glucose ([^18^F]FDG) in patients with unclear adrenal lesions, as well as confirmed ACC. This approach is also endorsed by current guidelines and reviews [5, 9–11]. Beyond accurate staging in indeterminate cases [12, 13], [^18^F]FDG reflects the metabolism of the tumor lesions and also provides the ability to quantify radiotracer accumulation in disease sites. This enables a *virtual biopsy* of every single lesion within the field-of-view [14]. The potential of the quantitatively derived PET signal in ACC patients has recently been evaluated in terms of the relationship between [^18^F]FDG uptake, clinicopathological and outcome data [15].

By investigating the up-to-date largest, long-term followed-up cohort of ACC patients imaged with [^18^F]FDG PET/CT prior to any guideline-directed treatment, we aimed to determine the predictive value of the [^18^F]FDG signal.

## Material and methods

### Patient characteristics

This retrospective single center study, 67 treatment-naïve patients with afterwards histologically confirmed ACC (*n* = 66) or metastatic ACC with tumor-induced autonomous hormone excess (*n* = 1) were included. They underwent [^18^F]FDG PET/CT prior to any ACC-related treatment between September 2011 and September 2022 (Supplementary Fig. 1). Other inclusion criteria were: age ≥18 years, available clinical/histopathological data and follow-up data (Table 1). Metastatic status was determined by pre-therapeutic [^18^F]FDG PET/CT performed as part of routine care according to current guidelines [5, 9]. In addition, the following clinical data were obtained from the ENSAT registry or our medical archive: sex, age at time of diagnosis, hormonal assessment, ENSAT stage, Weiss-Score and Ki-67 index of the primary tumor [5, 7, 16]. Tumor size was measured on the pre-treatment CT scan.

**Table 1:** Clinical and tumor characteristics

Patient characteristics	Entire cohort	M0 at time of diagnosis	M1 at time of diagnosis
*N*	67	24	43
Age at time of diagnosis in years,	50.5 ± 16.2 (18–79)	51.8 ± 15.7 (25–79)	49.7 ± 16.6 (18–79)
Sex (F:M)	40 (60%): 27 (40%)	14 (58%): 10 (42%)	26 (60%): 17 (40%)
Location of ACC (R:L)	27 (40%): 40 (60%)	10 (42%): 14 (58%)	17 (40%): 26 (60%)
**Tumor characteristics**			
Size, cm, mean ± SD Median (range)	9.9 ± 3.4; 9.7 (3.4–16.4)	8.7 ± 3.5; 9.1 (3.4–14.8)	10.6 ± 3.2; 10.6 (3.5–16.4)
HU in unenhanced CT, mean ± SD median (range)	33 ± 6; 33 (21–54)	35 ± 7; 34 (23–54)	33 ± 6; 33 (21–44)
Autonomous hormone excess	55 (83%) (1 not known)	17 (74%) (1 not known)	38 (88%)
**Histopathological parameters/ENSAT**			
Ki-67 Index, %	28.3 ± 18.7; 25 (1–90)	20.1 ± 18.9; 15 (1–80)	34.3 ± 16.4; 30 (10–90)
Weiss Score	6.7 ± 2; 7 (4–10)	6 ± 2; 6 (4–9)	7.3 ± 2; 8 (4–10)
ENSAT stage			
1	5 (7.5%)	5 (21%)	–
2	8 (12%)	8 (33%)	–
3	11 (16.5%)	11 (46%)	–
4	43 (64%)	–	43 (100%)
**Metastasized at initial diagnosis (M1)**	43/67 (64%)	–	43
In one organ compartment (M1-1)	19/67 (28%)	–	19/43 (44%)
In ≥2 organ compartments (M1-2)	24/67 (36%)	–	24/43 (56%)
**Location of metastases**	*n* = 77	–	*n* = 77
Lung	27 (35.1%)	**–**	27 (35.1%)
Liver	24 (31.2%)	–	24 (31.2%)
Lymph node	17 (22.1%)	–	17 (22.1%)
Bone	5 (6.5%)	–	5 (6.5%)
Other locations^a^	4 (5.1%)	–	4 (5.1%)
**Patients with additional findings in PET/CT compared to CT alone**^b^	8/67 (12%)	–	8/43 (19%)
**Therapy**			
Adrenalectomy	56 (84%)	24	32
Mitotane	45 (67%)	10	35
Chemotherapy	27 (40%)	3	24
Others	6 (9%)	1	5
**Median OS, months**	48 (1–139)	n.r. (9–136)	31 (1–139)
Dead	32/67 (44.8%)	2/24	30/43
Median PFS, months	11 (1–131)	37 (5–136)	7 (1–99)

Total amount or mean ± SD; median (range or percentages are indicated in parentheses)

*M0* patients with no evidence of metastases at initial diagnosis, *M1* patients with presence of metastatic disease at initial diagnosis, *OS* Overall survival, *PFS* progression-free survival, *ENSAT* European Network for the Study of Adrenal Tumors, *n.r.* not reached

^a^Including local recurrence, peritoneal and cerebral metastases

^b^Including mediastinal lymphnodes (*n* = 3), metastases located in bones or extremities (*n* = 2), liver (*n* = 2), vascular tumor invasion (*n* = 1)

The study was performed in accordance with the Declaration of Helsinki and the German Medical Products Act, AMG §13.2b. All patients provided written informed consent for the present retrospective data analysis, as they were included in the ENSAT registry. The local Ethics Committee waived the need for further approval because of the retrospective character of the study (waiver no. 20220519 03).

### Imaging procedures

Patients fasted for at least 6 h before image acquisition and their blood glucose levels were less than 160 mg/dL. A mean activity of 275.8 MBq (±51 MBq) [^18^F]FDG was injected intravenously. After 1 h, scanning was performed using a hybrid PET/CT scanner with an extended field-of-view for the PET and a 64- or 128-slice spiral CT (Biograph64 or 128, Siemens Healthineers; Erlangen, Germany). A whole-body PET scan covered the area from the skull to the upper thighs. Diagnostic CT scans were performed for attenuation correction and diagnostic purposes using the CT protocol with (*n* = 53) or without (*n* = 14) iodine contrast administration (depending on patient’s previous images). For CT-scans, automatic tube current modulation was activated, with reference mAs of 35 mAs for low-dose scans, and 160 mAs for full-dose scans. The tube voltage was set to 120 keV on the 64-slice CT-scanner, and 100 keV on the 128-slice CT-scanner. For collimation, we used 64/128 × 0.6 mm, while rotation time was 0.5 s. Axial slices were reconstructed with a thickness of 3.0 or 5.0 mm. After decay and scatter correction, the PET data underwent iterative reconstruction with attenuation correction, using the algorithm supplied by the scanner manufacturer using 3D mode with a 200 × 200 matrix, 3 iterations and 24 subsets for the mCT64 and 21 subsets for the mCT128. Additionally, Gaussian filtering of 2 mm was applied [17, 18].

### Visual and quantitative image interpretation

Tumor size of the adrenal tumor was measured according to the modified Response Evaluation Criteria in solid tumors (version 1.1) by using the trans-axial slice with the largest diameter [19]. Hounsfield Units (HU) of the primary tumor were measured on the unenhanced CT scan using a circular region of interest that include at least two-thirds of the lesion, carefully recessing the lesion’s margins to minimize partial volume effects [20].

**Fig. 1:**
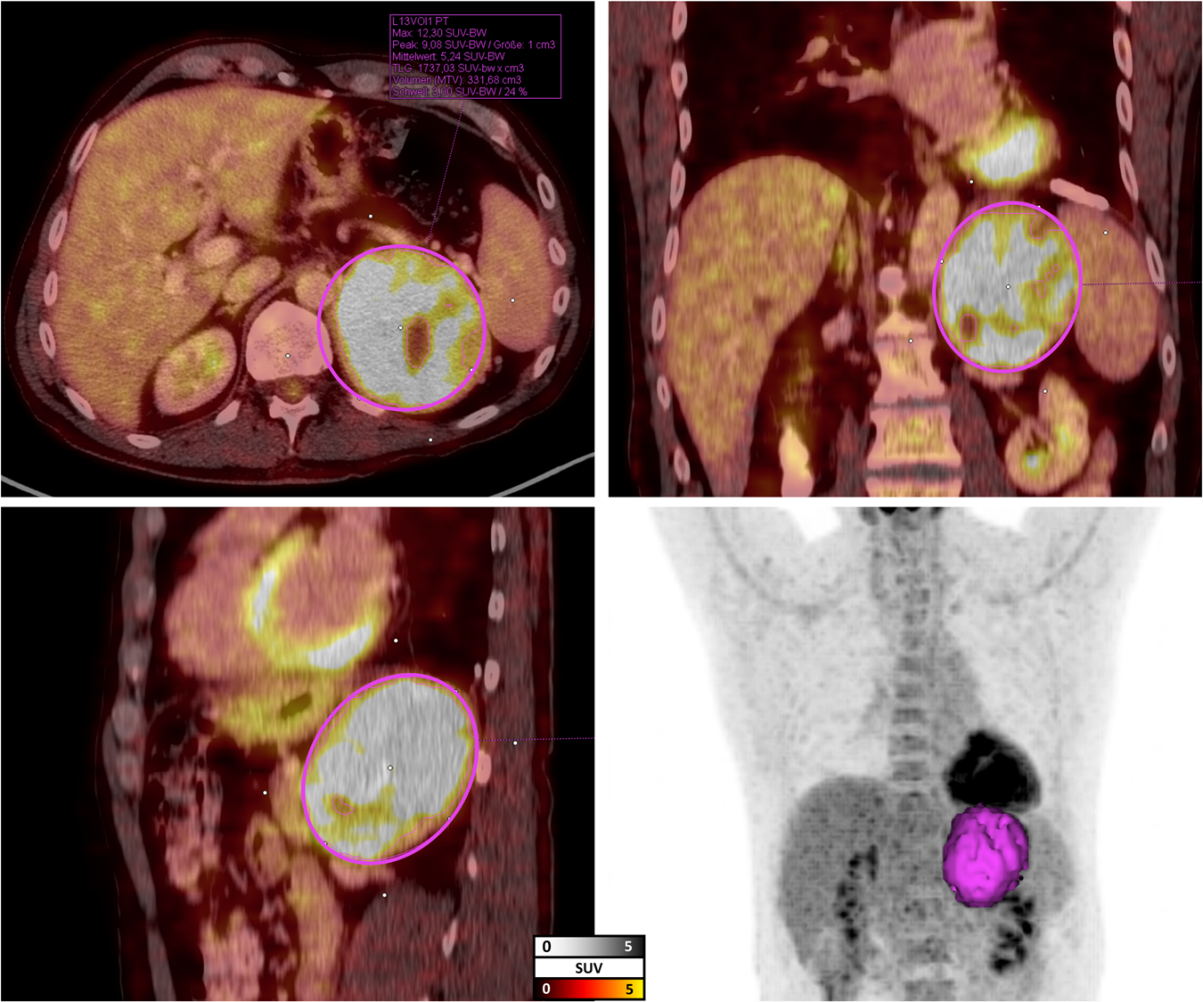
Example of segmentation of the primary tumor. An isocontour volume of interest (VOI) using a threshold SUV of 3.0 was drawn using a three-dimensional segmentation method allowing for a semi-automatic volumetric assessment (measurement results of the patient example: SUV_max_ 12.3. SUV_peak_ 9.08. SUV_mean_ 5.24. TLG 1737.03. MTV 331.68)

All PET/CT images were reviewed by a board-certified radiologist with three years of experience in reading PET/CT (W.S.) and supervised by a board-certified nuclear medicine physician (R.A.W.) using a dedicated workstation and software package (syngo.via; V60A; Siemens Healthineers, Erlangen, Germany). Based on a visual PET/CT readout, we defined the absence (M0) or presence of metastatic disease (M1), with M1-1 was defined as one and M1-2 as at least two affected organ compartments. Moreover, to quantify of the primary adrenal tumor, an isocontour volume of interest (VOI) with a threshold SUV of 3.0 was drawn using a three-dimensional segmentation method that allows semi-automatic volumetric assessment (Fig. 1) [17]. We calculated mean, maximum and peak standardized uptake values (SUV_mean/max/peak_). In addition, the metabolic tumor volume (MTV in cm^3^) of the primary adrenal tumor was also assessed, while tumor lesion glycolysis (TLG) was calculated using the following equation [21]:

1 $${{{\mathrm{TLG}}}} = {{{\mathrm{SUV}}}}_{{{{\mathrm{mean}}}}} \ast {{{\mathrm{MTV}}}}$$

Tumor-to-background ratios (TBR) were determined to quantify the image contrast and defined as follows:

2 $${{{\mathrm{TBR}}}} = {{{\mathrm{SUV}}}}_{{{{\mathrm{max}}}}}\left( {{{{\mathrm{of}}}}\;{{{\mathrm{the}}}}\;{{{\mathrm{adrenal}}}}\;{{{\mathrm{tumor}}}}} \right){{{\mathrm{/SUV}}}}_{{{{\mathrm{mean}}}}}\left( {{{{\mathrm{unaffected}}}}\;{{{\mathrm{liver}}}}\;{{{\mathrm{parenchyma}}}}} \right)$$

As previously used in several studies, we also determined the Adrenal-to-liver SUV_max_ ratio (ALR) [15, 22, 23]:

3 $${{{\mathrm{ALR}}}} = {{{\mathrm{SUV}}}}_{{{{\mathrm{max}}}}}\left( {{{{\mathrm{of}}}}\;{{{\mathrm{the}}}}\;{{{\mathrm{adrenal}}}}\;{{{\mathrm{tumor}}}}} \right){{{\mathrm{/SUV}}}}_{{{{\mathrm{max}}}}}\left( {{{{\mathrm{unaffected}}}}\;{{{\mathrm{liver}}}}\;{{{\mathrm{parenchyma}}}}} \right)$$

### Statistical analysis

For statistical analysis, GraphPad Prism (GraphPad Software, version 9.4.1, San Diego, CA, USA) was used. Continuous variables were presented as mean ± SD or median and range, as appropriate. Mann–Whitney *U* test was used to compare groups regarding continuous variables. Overall survival (OS) was defined as time from first diagnosis to death or last follow-up and progression-free survival (PFS) was scored as time interval from first day of treatment to first documented disease progression. Kaplan–Meier survival curves were calculated using the median of the parameter to illustrate separation. Uni- and multivariable Cox regressions were used for survival prediction and to identify independent prognostic factors. Hazard ratio (HR) along with 95% confidence intervals (*95% CI*) are displayed. Spearman rank order correlation test was used to assess correlation between [^18^F]FDG PET parameter and Ki-67. *P* < 0.05 was considered statistically significant.

## Results

The detailed characteristics of the patients are summarized in Table 1 and the flowchart of the study design is shown in Supplementary Fig. 1. Briefly, the entire cohort consisted of 67 patients (mean age 50.5 ± 16.2 years), of whom 40 (60%) were female. In 43/67 (64%), ENSAT stage was IV. A Ki-67 index ≥20% was observed in 35/56 (63%) of cases, while 26/47 (55%) had a Weiss-Score ≥7. According to PET/CT findings, 43/67 patients (64%) had metastatic disease (M1) (ENSAT stage IV), with 19/43 (44%) having one affected organ compartment (M1-1) and 24/43 (56%) having two affected organ compartments (M1-2). Compared to CT alone [^18^F]FDG PET/CT detected additional metastatic lesions in eight patients (8/43) (19%). [^18^F]FDG PET/CT, treatment included adrenalectomy in 56/67 (84%), mitotane in 45/67 (67%), and chemotherapy in 27/67 (40%). Tumor characteristics of the primary tumors are shown in Tables 1 and 2. The median follow-up of surviving patients was 40 months. Median PFS and OS were 10 and 48 months, respectively. 32/67 patients (45%) died during follow-up.

**Table 2:** Quantitative analyses of entire cohort and M0/M1-group for [^18^F]FDG PET

		Entire cohort	M0	M1	M0 vs. M1
*N*			24	43	*p*-value
SUV_peak_	Mean ± SD	15.9 ± 11.3	13.7 ± 13.2	22.4 ± 10.2	
	Median (range)	12.4 (3–57)	10 (3–57)	14 (7–51)	<0.05
SUV_max_	Mean ± SD	20.6 ± 14.5	17.2 ± 16	22.4 ± 13.5	
	Median (range)	17.1 (4–71)	11.8 (4–71)	17.5 (8–71)	<0.05
SUV_mean_	Mean ± SD	7.0 ± 3.8	6.4 ± 4.3	7.4 ± 3.4	
	Median (range)	6.2 (3–22)	5.5 (3–22)	6.5 (4–21)	<0.05
TLG	Mean ± SD	3629 ± 5562	2265 ± 2545	4391 ± 6588	
	Median (range)	2007 (25–42545)	1579 (25–9469)	2231 (144–42545)	<0.05
MTV	Mean ± SD	470 ± 469	336 ± 323	545 ± 522	
	Median (range)	319 (8–2952)	258 (8–1205)	348 (25–2952)	0.06
TBR	Mean ± SD	9.2 ± 7.5	7.6 ± 7.7	10.1 ± 7.3	
	Median (range)	6.6 (2–33)	4.9 (2- 31)	7.6 (4– 33)	<0.05
Adrenal-to-liver SUV_max_ ratio	Mean ± SD	6.2 ± 4.9	5.3 ± 5.5	6.7 ± 4.5	<0.01
	Median (range)	4.8 (1.1–23.6)	3.6 (1.1–23.0)	5.0 (2.8–23.6)	

*M0* patients with no evidence of metastases at initial diagnosis, *M1* patients with presence of metastatic disease at initial diagnosis, Comparison of [^18^F]FDG PET-parameters of *M0* and *M1* using Mann–Whitney *U* test, *SUV* standardized uptake value, *TLG* tumor lesion glycolysis, *MTV* metabolic tumor volume, *TBR* target to background ratio

All ACC presented with high SUVs: The lowest adrenal SUV_max_ was 4.2 in a patient with an incidentally discovered ACC (M0, 44 mm diameter) and all other patients showed SUV_max_ 6.0 or higher. Dividing the cohort into patients with (M1) and without (M0) presence of metastases at initial diagnosis, SUV_peak/max/mean_, TLG, TBR and ALR were significant different with higher values in the patient group with M1-status (*P* < 0.05) (Table 2).

### Associations between quantitative PET results and clinical outcome

**Fig. 2:**
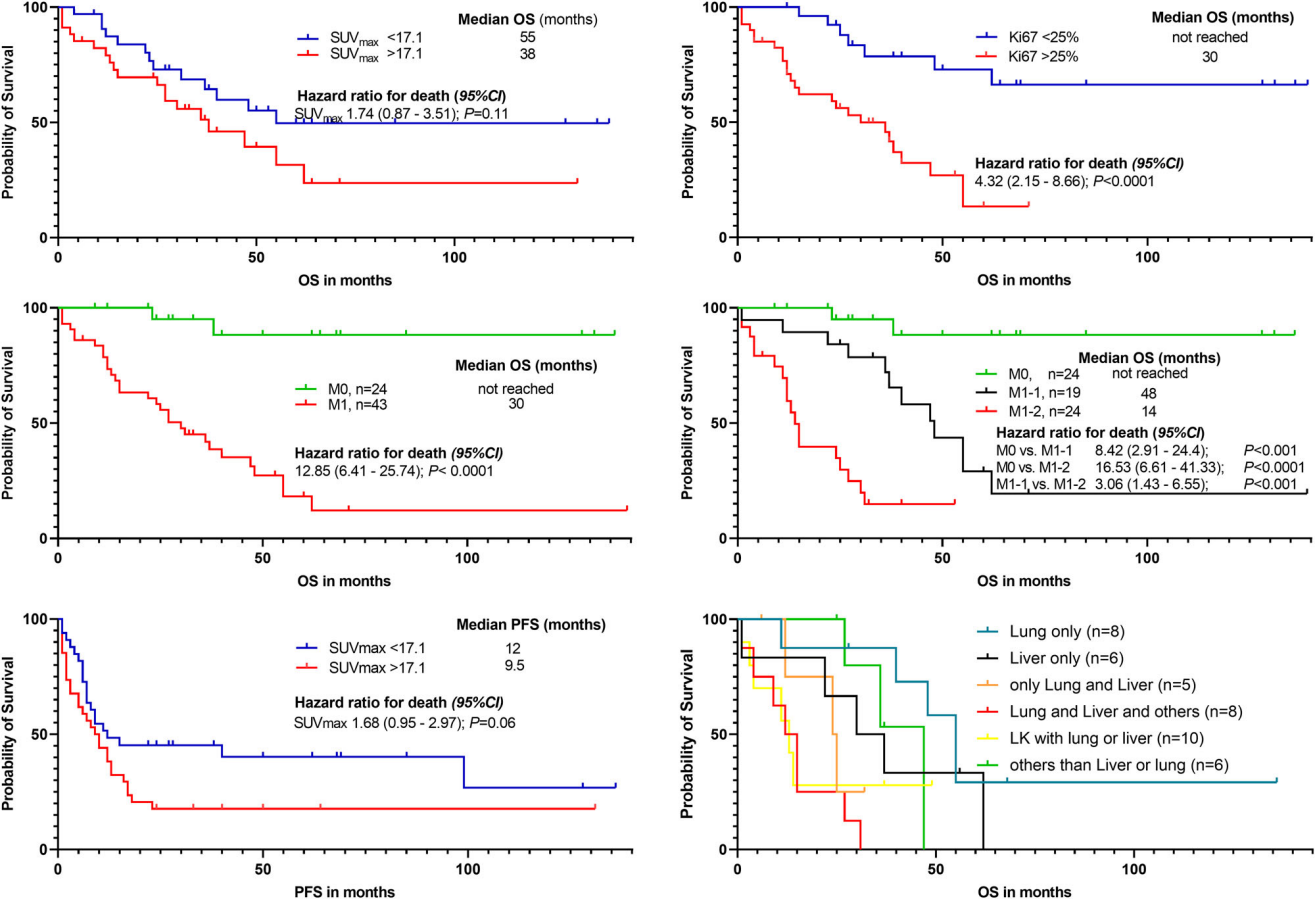
Kaplan–Meier plots for probability of progression free and overall survival (PFS/OS) using SUV_max_, TBR and Ki-67 index, as well as presence of metastatic disease (M1) based on [^18^F]FDG PET/CT and number of organ compartments affected by metastases. In this regard, two or more affected organ compartments (M1-2) exhibited shortest survival relative to M1-1, defined as only one affected compartment. Regarding the localization of affected organ compartment, presence of liver metastases is linked to shorter survival (22 months) compared to presence of lung metastases (25 months)

**Fig. 3:**
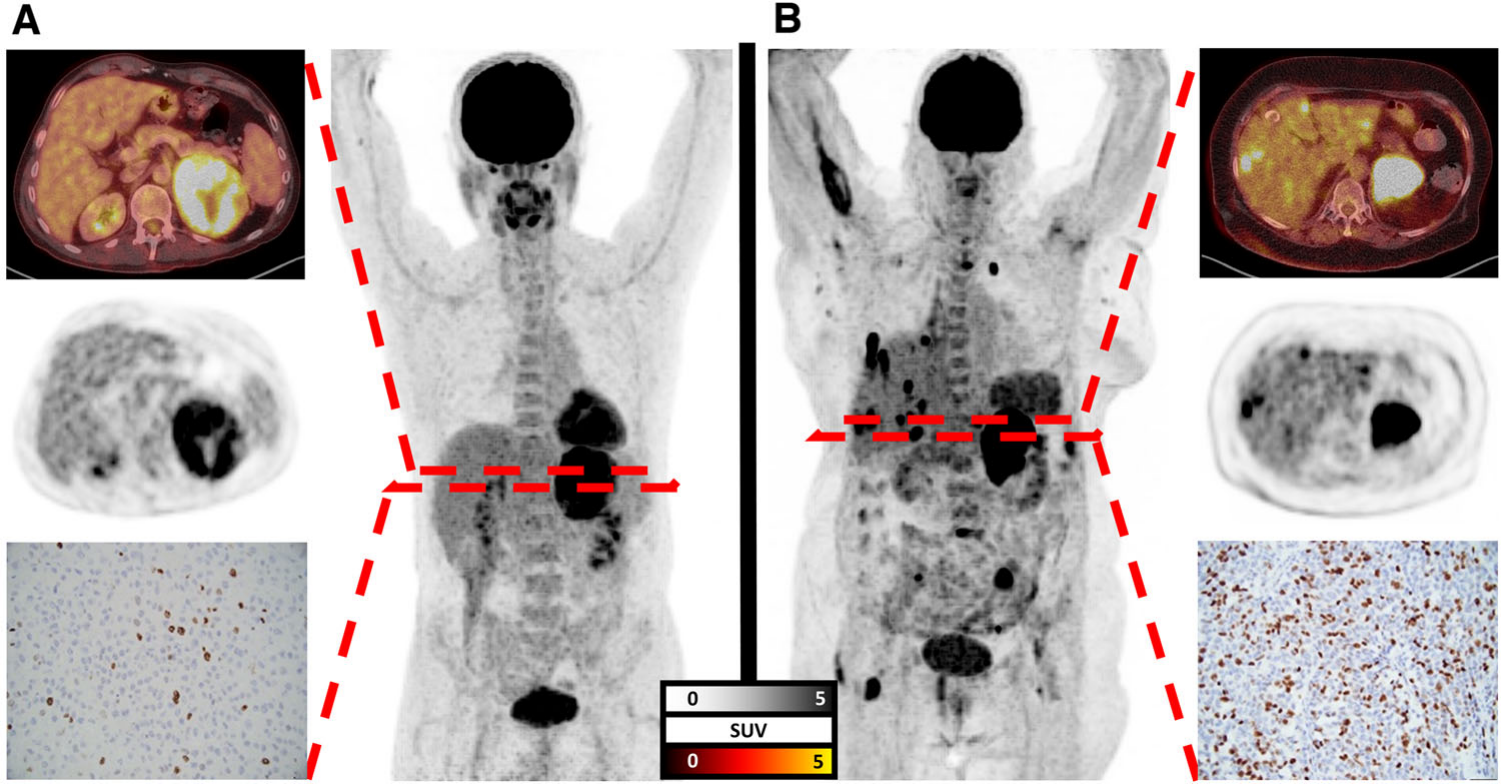
Pretherapeutic [^18^F]FDG PET/CT with maximum intensity projections in the middle, transaxial PET/CT and PET, along with immunohistochemistry revealing proliferation index (Ki-67). **A** 61 year-old male with primary located on the left side, but without metastatic spread. Ki-67 was 10%, i.e. under the median of 24.5%. As such, findings on PET/CT and proliferation index were indicative for prolonged survival. During follow-up, this patient was still alive 53 months after initial diagnosis. **B** 56 year-old female with primary located on the left side, along with metastases in lung, liver, lymph nodes and bone, i.e. four affected organ compartments derived from PET/CT. Histopathology revealed a Ki-67 of 50%, i.e. above the median of 27.5%. Taken together, immunohistochemistry and findings on pretherapeutic [^18^F]FDG PET/CT were indicative for shorter survival and relative to the patient presented in **A**, this subject succumbed to disease already 18 months after initial diagnosis

In Kaplan-Meier analyses, none of the quantitative PET parameters were associated with PFS or OS, although there was a trend towards improved PFS for SUV_max_ values < 17.1 (HR 1.68, 95% CI 0,95–2,97, *p* = 0.06). The PET/CT-based metastatic status was associated with shorter survival (M1, median OS, 30 months vs. M0, not reached; HR 12.85, 95% CI 6.41–25.74, *P* < 0.0001). Specifically, a higher number of organ compartments involved correlated negatively with OS: patients with M1-1 had a median OS of 48 months and patients with M1-2 survived only 14 months (Fig. 2). Regarding the proliferation index Ki-67, a higher Ki-67 correlated with shorter OS (Ki-67 > 25%, median OS 30 months vs. Ki-67 < 25%, not reached; *HR* 4.32, *95% CI* 2.15–8.66, *P* < 0.0001) (Fig. 2). Figure 3 presents two illustrative cases with treatment-naïve ACC with corresponding findings on immunohistochemistry and [^18^F]FDG PET/CT.

In univariable Cox regression analysis for OS, quantitatively derived PET and CT parameters, failed to reach significance (*P* ≥ 0.09; Table 3). Presence of metastases (M1) at initial diagnosis (*HR* 13.89, *95% CI* 4.15–86.32, *P* < 0.001) and an increase in Ki-67 (per 5%, *HR* 1.29, *95% CI* 1.16–1.42, *P* < 0.0001) were the only variables that were significantly associated with shorter overall survival. In multivariable Cox regression analyses only M1-status (*HR* 2.27, *95% CI* 1.04–4.11; *P* < 0.01) and Ki-67 (*HR* 1.29, *95% CI* 1.13–1.47; *P* < 0.001) remained significant (Table 3). In univariable Cox regression analyses for PFS only higher Ki-67 was associated with shorter PFS, while all other parameters failed to reach statistical significance (Table 4).

**Table 3:** Univariable and multivariable Cox regressions for overall survival

	Univariable			Multivariable		
	*HR*	*95% CI*	*P-value*	*HR*	*95% CI*	*P-value*
PET and CT parameters:						
Ki-67 index (per 5%)	1.285	1.160–1.423	**<0.0001**	1.294	1.134–1.039	**<0.0001**
M1	13.89	4.147–86.32	**0.0003**	9.690	2.815–60.99	**0.0023**
ENSAT stage	3.189	1.569–10.82	0.06	1.403	0.315–10.17	0.68
Weiss-Score	1.311	1.024–1.726	0.09			
TLG	1.000	1.000–1.000	0.09			
HU on unenhanced CT	0.947	0.881–1.017	0.14			
MTV	1.000	0.9997–1.001	0.17			
SUV_max_	1.011	0.987–1.031	0.31			
Tumor size	1.005	0.995–1.016	0.31			
TBR	1.019	0.972–1.060	0.40			
SUV_peak_	1.010	0.978–1.036	0.49			
SUV_mean_	1.027	0.939–1.110	0.55			
ALR	1.019	0.945–1.083	0.58			
Metastases in:						
Liver	5.357	2.609–11.23	**<0.0001**	4.741	2.211–10.4	**<0.0001**
Lung	3.379	1.665–7.159	**0.0009**	2.487	1.122–5.649	**0.0258**
Lymphnodes	3.290	1.482–6.999	**0.0024**	1.816	0.777–4.154	0.19
Bone	3.722	1.242–9.115	**0.0085**			

Significant *P*-values are marked in bold

*HR* Hazard ratio, *CI* confidence interval. *M1* presence of metastases at first diagnosis, *ENSAT* European Network for the Study of Adrenal Tumors, *TLG* tumor lesion glycolysis, *MTV* metabolic tumor volume, *SUV* standardized uptake value, *TBR* target to background (Liver) ratio, *ALR* adrenal to liver SUV_max_ ratio

**Table 4:** Univariable and multivariable Cox regressions for progression-free survival

	Univariable			Multivariable		
	*HR*	*95% CI*	*P-value*	*HR*	*95% CI*	*P-value*
Ki-67 index (per 5%)	1.035	0.996–1.069	**0.04**	1.018	0.938–1.072	0.55
HU on unenhanced CT	1.089	0.914–1.267	0.29	1.070	0.871–1.26	0.45
MTV	0.999	0.995–1.001	0.46			
TLG	0.999	0.998–1.000	0.49			
Tumor size	0.994	0.967–1.019	0.64			
TBR Liver	0.990	0.798–1.095	0.89			
SUV_peak_	0.996	0.881–1.055	0.93			
ALR	1.008	0.779–1.149	0.93			
SUV_mean_	1.009	0.725–1.185	0.93			
SUV_max_	1.001	0.916–1.048	0.96			
Weiss-Score	1.005	0.615–1.562	0.98			

Significant *P*-values are marked in bold

*HR* Hazard ratio, *CI* confidence interval, *M1* presence of metastatic disease, *TLG* tumor lesion glycolysis (defined as tumor volume × SUV_mean_), *MTV* metabolic tumor volume, *SUV*_peak/mean/max_ peak/mean/maximum Standard uptake value, *TBR* tumor-background ratio

Regarding the location of metastases at time of first diagnosis, presence of liver metastases is more indicative for a worse prognosis than lung metastases. In Kaplan–Meier analyses, combination of both and in addition other locations, e.g. lymph node metastases, are connected with shortest OS (Fig. 2). In univariable Cox regression analyses presence of liver and then lung metastases was linked to shorter survival (HR 5.36, 95% CI 1.67–7.16, *P* < 0.0001; respectively HR 3.38, 95% CI 1.67–7.16, *P* < 0.001) (Table 3).

## Discussion

As reported in previous studies, the ability of [18F]FDG PET/CT to discern between benign and malignant adrenal lesions ranges from 85% to 100% for both sensitivity and specificity [22, 23]. Nevertheless, the prognostic value of [18F]FDG PET/CT at the time of diagnosis of ACC has received little attention. In a previous study, we highlighted glucose transporter 1 (GLUT1) as a stage-independent prognostic biomarker for ACC, determined by assessing GLUT1 expression in tissue sections from ACC patients [24]. At the cellular level, the uptake of [18F]FDG is facilitated by GLUT1. Consequently, our investigation focused on this radiotracer reflecting glucose consumption in ACC patients before treatment, with the aim of validating its prognostic value.

In our study none of the quantitative PET parameters was of prognostic value, but in 8 patients (12%), additional metastases, not identified by CT, were detected by FDG-PET. Using multivariate regression analyses only the presence of [18F]FDG PET/CT-positive metastases and a higher Ki-67 index were associated with shorter OS. Thus, a simple visual PET-based read-out is probably as good as the time-consuming quantification of different sophisticated PET parameters in ACC.

A correlation between adrenal SUV_max_ and the proliferation marker Ki-67 has recently been described by Libé et al. [15]. In our study, we are able to confirm these findings. Intriguingly, individuals with metastases at time of initial diagnoses had significantly elevated quantitative PET parameters in their primary tumor (except MTV) compared to those without metastases. The clinical implications of this observation need be evaluated in a prospective study.

Considering outcome data, Takeuchi et al. showed that SUV_max_ and TLG were also not associated with survival, but their study cohort with [^18^F]FDG PET/CT for primary staging consisted of only 22 patients with ACC [25]. Beyond such conventional metrics, a previously published pilot study added radiomics to the quantitative armamentarium, but these PET-based mathematically extracted features also failed to predict outcome [14]. While these analyses included a rather limited number of subjects, Tessonnier et al. investigated the prognostic value of pre-therapeutic PET/CT in 37 patients and also did not report on predictive capabilities of SUV_max_ or tumor/liver SUV_max_ ratio (ALR) [24].

As initially described by Tessonnier et al., an ALR of 1.8 demonstrated 100% sensitivity and specificity for differentiating between benign and malignant adrenal tumors, but their study cohort was heterogeneous and only 3 ACC were included [22]. Later this ALR value was slightly modified by Groussin et al. after prospective evaluation of the role of [^18^F]FDG PET/CT excluding pheochromocytoma and other non-adrenal malignancies. A ratio below 1.45 showed the best negative predictive value for ACC [23]. Using these ratios on our cohort we would have missed two ACCs (ALR of 1.12, M0, 44 mm diameter and ALR of 1.4, M0, 49 mm diameter). Comparing ALR and TBR (= SUV_max_ adrenal/SUV_mean_ liver) which is more established in clinical nuclear medicine routine as it is the more stable and meaningful parameter [21], we see comparable results regarding the differentiation between patients with and without metastases and no significant association between survival parameters (OS and PFS).

In the present single-center experience, with the largest number of treatment-naïve ACC patients an increasing number of affected organ compartments (≥2) was associated with shorter OS. This is in contrast to the results of Leboulleux et al. who performed a [^18^F]FDG PET/CT study in a cohort of 28 mainly metastatic patients after various previous treatment options at a median of 3 years after ACC tumor surgery. In their study the number of affected organs (>2) did not determine the prognosis, but in univariable analysis SUV_max_ > 10 was associated with decreased survival [26]. The differences to our study may be related to previous treatments. We only included patients who had not received any anti-cancer-related treatment. This ensures that the molecular and biological characteristics of the tumor tissue were not affected by treatment-induced dedifferentiation, which could otherwise lead to increased expression of glucose transporters and glycolytic enzymes. Consequently, this could result in an elevated FDG-PET signal.

Regarding the location of metastases, Ettaieb et al. found that OS was not different between their subgroups with only one affected organ system [27]. In our cohort, the presence of liver metastases was associated with a worse outcome than the presence of lung metastases, but the small number of these subgroups limits the significance of this observation. Compared to this a visual assessment of involved organs, time-consuming segmentation and quantification of glucose consumption in the primary failed to reach significance. As such, a simple PET-based read-out of affected organ compartments may be sufficient to identify individuals with a less favorable outcome, thereby rendering molecular imaging as a valuable tool for identifying patients who may benefit from intensified treatment early in the course of the disease.

Of note, in our study investigating PET/CTs prior to treatment on-set, HU derived from conventional unenhanced CT failed to reach significance even in univariable analysis.

However, novel artificial intelligence approaches may also enable for providing sufficient number of PET/CTs, as those Deep Convolutional Generative Adversarial Networks require only a relatively small number of existing scans to create novel images that closely resemble their real-world equivalents. Such a deep learning-based augmentation, however, may be of particular interest in the context of orphan diseases such as ACC or in scenarios chosen in the present study, which focused exclusively on untreated subjects [28]. Last, a recent study investigated 690 patients affected with 35 different tumor types, which were all imaged with chemokine receptor PET. In the subgroup of solid cancers, ACC showed the highest in vivo uptake. Thus, future studies may also determine the value of such novel radiotracers for outcome prediction in treatment-naïve ACC patients [29].

Our study has some obvious limitations. First, it is limited by its retrospective nature. Secondly, the sample size is still rather small. Regarding the patient cohort itself, the large number of patients with metastases at time of initial diagnosis, resulting in a large group with ENSAT stage IV, could be a limitation as they often show a more unfavorable outcome. This can be attributed to the fact that advanced patients were sent to our specialized ACC center, whereas non-metastatic patients were often operated in peripheral hospitals without preoperative molecular imaging. As such, future prospective studies should also include more patients with ENSAT stage I and II. However, our study also has strengths: the detailed characterization of all PET/CT images by a board-certified radiologist and a board-certified nuclear medicine physician, the comprehensive clinical annotation of the patient cohort, and long-term follow-up of our study cohort.

## Conclusion

In this largest analysis to date of treatment-naïve ACC patients scheduled for [^18^F]FDG PET/CT, quantitative PET-parameters such as SUV_peak/max/mean_, tumor-to-background ratio failed to predict OS. However, a higher SUV_peak/max/mean_ of the primary tumor correlated with presence of metastases and might identify patients that deserve special attention in the search for metastases. Accordingly, in 12% of patients FDG-PET detected a metastatic lesion not clearly visible by CT alone. Furthermore, molecular imaging-based M1-status was independent of other prognostic markers associated to shorter OS, especially when two or more organ compartments were involved. As such, a simple read-out of affected organ compartments may be sufficient to identify patients at higher risk of shorter survival, making molecular imaging a valuable tool for identifying patients who may benefit from intensified treatment early in the course of their disease.

### Supplementary information

Supplementary Figure 1

Supplementary Figure legend

## Data Availability

Detailed information about the image analysis or the overall survivals of the subjects presented in this study are available on reasonable request from the corresponding author.

## References

[1] Kebebew E, Reiff E, Duh QY, Clark OH, McMillan A (2006). Extent of disease at presentation and outcome for adrenocortical carcinoma: have we made progress?. World J. Surg..

[2] Kerkhofs TM, Verhoeven RH, Van der Zwan JM, Dieleman J, Kerstens MN (2013). Adrenocortical carcinoma: a population-based study on incidence and survival in the Netherlands since 1993. Eur. J. Cancer.

[3] Fassnacht M, Johanssen S, Quinkler M, Bucsky P, Willenberg HS (2009). Limited prognostic value of the 2004 International Union Against Cancer staging classification for adrenocortical carcinoma: proposal for a Revised TNM Classification. Cancer.

[4] Else T, Williams AR, Sabolch A, Jolly S, Miller BS (2014). Adjuvant therapies and patient and tumor characteristics associated with survival of adult patients with adrenocortical carcinoma. J. Clin. Endocrinol. Metab..

[5] Fassnacht M, Dekkers OM, Else T, Baudin E, Berruti A (2018). European society of endocrinology clinical practice guidelines on the management of adrenocortical carcinoma in adults, in collaboration with the European Network for the study of adrenal tumors. Eur. J. Endocrinol..

[6] Lughezzani G, Sun M, Perrotte P, Jeldres C, Alasker A (2010). The European Network for the Study of Adrenal Tumors staging system is prognostically superior to the international union against cancer-staging system: a North American validation. Eur. J. Cancer.

[7] Libe R, Borget I, Ronchi CL, Zaggia B, Kroiss M (2015). Prognostic factors in stage III-IV adrenocortical carcinomas (ACC): an European Network for the Study of Adrenal Tumor (ENSAT) study. Ann. Oncol..

[8] Elhassan YS, Altieri B, Berhane S, Cosentini D, Calabrese A (2021). S-GRAS score for prognostic classification of adrenocortical carcinoma: an international, multicenter ENSAT study. Eur. J. Endocrinol..

[9] Fassnacht M, Assie G, Baudin E, Eisenhofer G, de la Fouchardiere C (2020). Adrenocortical carcinomas and malignant phaeochromocytomas: ESMO–EURACAN Clinical Practice Guidelines for diagnosis, treatment and follow-up†. Ann. Oncol..

[10] Kim SJ, Lee SW, Pak K, Kim IJ, Kim K (2018). Diagnostic accuracy of (18)F-FDG PET or PET/CT for the characterization of adrenal masses: a systematic review and meta-analysis. Br. J. Radio..

[11] Fassnacht M, Tsagarakis S, Terzolo M, Tabarin A, Sahdev A (2023). European Society of Endocrinology Clinical Practice Guidelines on the management of adrenal incidentalomas, in collaboration with the European Network for the Study of Adrenal Tumors. Eur. J. Endocrinol..

[12] Nakajo M, Jinguji M, Nakajo M, Shinaji T, Nakabeppu Y (2017). Texture analysis of FDG PET/CT for differentiating between FDG-avid benign and metastatic adrenal tumors: efficacy of combining SUV and texture parameters. Abdom. Radio..

[13] Guerin C, Pattou F, Brunaud L, Lifante JC, Mirallie E (2017). Performance of 18F-FDG PET/CT in the Characterization of Adrenal Masses in Noncancer Patients: A Prospective Study. J. Clin. Endocrinol. Metab..

[14] Werner RA, Kroiss M, Nakajo M, Mugge DO, Hahner S (2016). Assessment of tumor heterogeneity in treatment-naive adrenocortical cancer patients using (18)F-FDG positron emission tomography. Endocrine.

[15] Libe R, Pais A, Violon F, Guignat L, Bonnet F (2023). Positive Correlation Between 18 F-FDG Uptake and Tumor-Proliferating Antigen Ki-67 Expression in Adrenocortical Carcinomas. Clin. Nucl. Med.

[16] Ronchi CL, Sbiera S, Leich E, Tissier F, Steinhauer S (2012). Low SGK1 expression in human adrenocortical tumors is associated with ACTH-independent glucocorticoid secretion and poor prognosis. J. Clin. Endocrinol. Metab..

[17] Boellaard R, Delgado-Bolton R, Oyen WJ, Giammarile F, Tatsch K (2015). FDG PET/CT: EANM procedure guidelines for tumour imaging: version 2.0. Eur. J. Nucl. Med Mol. Imaging.

[18] Kosmala A, Serfling SE, Schlotelburg W, Lindner T, Michalski K (2023). Impact of 68 Ga-FAPI-04 PET/CT on Staging and Therapeutic Management in Patients With Digestive System Tumors. Clin. Nucl. Med..

[19] Schwartz LH, Litiere S, de Vries E, Ford R, Gwyther S (2016). RECIST 1.1-Update and clarification: From the RECIST committee. Eur. J. Cancer.

[20] Schloetelburg W, Ebert I, Petritsch B, Weng AM, Dischinger U (2021). Adrenal wash-out CT: moderate diagnostic value in distinguishing benign from malignant adrenal masses. Eur. J. Endocrinol..

[21] Wahl RL, Jacene H, Kasamon Y, Lodge MA (2009). From RECIST to PERCIST: Evolving Considerations for PET response criteria in solid tumors. J. Nucl. Med.

[22] Tessonnier L, Sebag F, Palazzo FF, Colavolpe C, De Micco C (2008). Does 18F-FDG PET/CT add diagnostic accuracy in incidentally identified non-secreting adrenal tumours?. Eur. J. Nucl. Med Mol. Imaging.

[23] Groussin L, Bonardel G, Silvera S, Tissier F, Coste J (2009). 18F-Fluorodeoxyglucose positron emission tomography for the diagnosis of adrenocortical tumors: a prospective study in 77 operated patients. J. Clin. Endocrinol. Metab..

[24] Tessonnier L, Ansquer C, Bournaud C, Sebag F, Mirallie E (2013). (18)F-FDG uptake at initial staging of the adrenocortical cancers: a diagnostic tool but not of prognostic value. World J. Surg.

[25] Takeuchi S, Balachandran A, Habra MA, Phan AT, Bassett RL (2014). Impact of (1)(8)F-FDG PET/CT on the management of adrenocortical carcinoma: analysis of 106 patients. Eur. J. Nucl. Med Mol. Imaging.

[26] Leboulleux S, Dromain C, Bonniaud G, Auperin A, Caillou B (2006). Diagnostic and prognostic value of 18-fluorodeoxyglucose positron emission tomography in adrenocortical carcinoma: a prospective comparison with computed tomography. J. Clin. Endocrinol. Metab..

[27] Ettaieb MH, Duker JC, Feelders RA, Corssmit EP, Menke-van der Houven van Oordt CW (2016). Synchronous vs. Metachronous Metastases in Adrenocortical Carcinoma: an Analysis of the Dutch Adrenal Network. Horm. Cancer.

[28] Werner RA, Higuchi T, Nose N, Toriumi F, Matsusaka Y (2022). Generative adversarial network-created brain SPECTs of cerebral ischemia are indistinguishable to scans from real patients. Sci. Rep..

[29] Buck AK, Haug A, Dreher N, Lambertini A, Higuchi T (2022). Imaging of C-X-C Motif Chemokine Receptor 4 Expression in 690 Patients with Solid or Hematologic Neoplasms Using (68)Ga-Pentixafor PET. J. Nucl. Med..

