# Supplementary figures and images for: Prognostic role of quantitative [18F]FDG PET/CT parameters in adrenocortical carcinoma
Source: Endocrine. 2024 Feb 21;84(3):1172–81. doi: 10.1007/s12020-024-03695-6 (PMC11208261; doi:10.1007/s12020-024-03695-6)

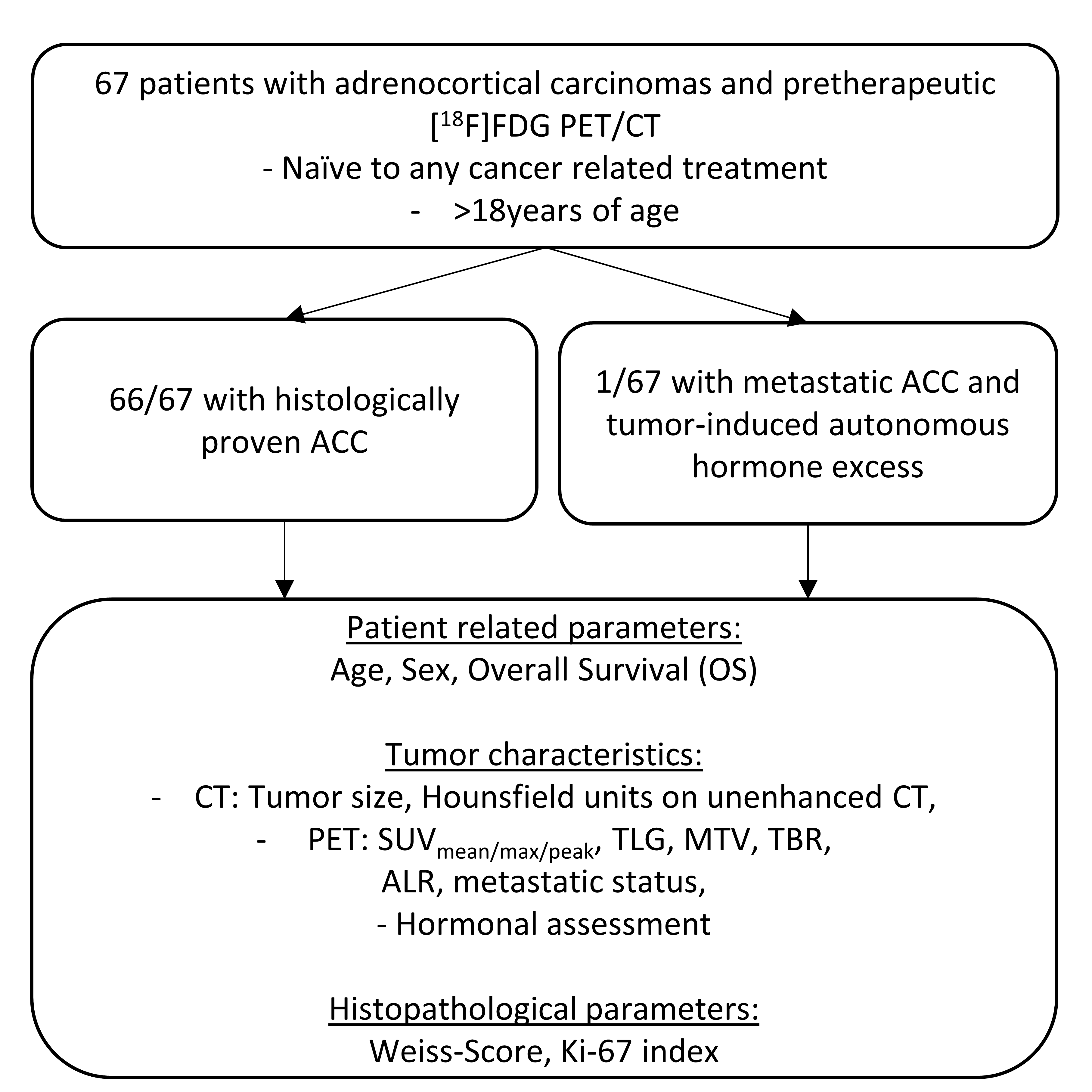

Supplement: Supplementary file 1 — Supplementary Figure 1 [file 12020_2024_3695_MOESM1_ESM.tif]
