# Supplementary material for: Prognostic role of quantitative [18F]FDG PET/CT parameters in adrenocortical carcinoma
Source: Endocrine. 2024 Feb 21;84(3):1172–81. doi: 10.1007/s12020-024-03695-6 (PMC11208261; doi:10.1007/s12020-024-03695-6)
Supplement: Supplementary file 2 — Supplementary Figure legend [file 12020_2024_3695_MOESM2_ESM.docx]

**Suppl. Fig. 1** Flowchart of study design. Patient population and analysis description. SUV, standardized uptake value. TLG, tumor lesion glycolysis. MTV, metabolic tumor volume. TBR, target to background (Liver) ratio. ALR, adrenal to liver SUV_max_ ratio.
